# The Other Side of SARS-CoV-2 Infection: Neurological Sequelae in Patients

**DOI:** 10.3389/fnagi.2021.632673

**Published:** 2021-04-06

**Authors:** Isabel M. Alonso-Bellido, Sara Bachiller, Guillermo Vázquez, Luis Cruz-Hernández, Emilio Martínez, Ezequiel Ruiz-Mateos, Tomas Deierborg, José L. Venero, Luis M. Real, Rocío Ruiz

**Affiliations:** ^1^Departamento de Bioquímica y Biología Molecular, Facultad de Farmacia, Universidad de Sevilla, Sevilla, Spain; ^2^Instituto de Biomedicina de Sevilla-Hospital, Universitario Virgen del Rocío/CSIC/Universidad de Sevilla, Sevilla, Spain; ^3^Experimental Neuroinflammation Laboratory, Department of Experimental Medical Science, Biomedical Center, Lund University, Lund, Sweden; ^4^Unidad Clínica de Enfermedades Infecciosas, Microbiología y Medicina Preventiva, Instituto de Biomedicina de Sevilla-Hospital Universitario Virgen del Rocío/CSIC/Universidad de Sevilla, Sevilla, Spain; ^5^Unidad Clínica de Enfermedades Infecciosas y Microbiología, Hospital Universitario de Valme, Sevilla, Spain; ^6^Departamento de Especialidades Quirúrgicas, Bioquímicas e Inmunología, Facultad de Medicina, Universidad de Málaga, Málaga, Spain

**Keywords:** coronavirus, COVID-19, SARS-CoV-2, neurological, nervous system

## Abstract

The severe acute respiratory syndrome coronavirus 2 (SARS-CoV-2) has spread around the globe causing coronavirus disease 2019 (COVID-19). Because it affects the respiratory system, common symptoms are cough and breathing difficulties with fever and fatigue. Also, some cases progress to acute respiratory distress syndrome (ARDS). The acute phase of COVID-19 has been also related to nervous system symptoms, including loss of taste and smell as well as encephalitis and cerebrovascular disorders. However, it remains unclear if neurological complications are due to the direct viral infection of the nervous system, or they appear as a consequence of the immune reaction against the virus in patients who presented pre-existing deficits or had a certain detrimental immune response. Importantly, the medium and long-term consequences of the infection by SARS-CoV-2 in the nervous system remain at present unknown. This review article aims to give an overview of the current neurological symptoms associated with COVID-19, as well as attempting to provide an insight beyond the acute affectation.

## Introduction

The severe acute respiratory syndrome coronavirus 2 (SARS-CoV-2) has rapidly caused a pandemic, only a few months after the first cases reported in December 2019. Although some infected individuals are asymptomatic, manifestations of the SARS-CoV-2 disease (coronavirus disease 2019, COVID-19) are cough, breathing difficulty (dyspnea) with fever, and fatigue (asthenia). However, some cases show severe bilateral pneumonia and progress to acute respiratory distress syndrome (ARDS) and multiorgan failure (Chen et al., 2020; Dhama et al., 2020; Huang et al., 2020; Zhang J. J. Y. et al., 2020).

Recent works have described neurological manifestations that ranged from mild to fatal in both asymptomatic and symptomatic patients infected by SARS-CoV-2 (Helms et al., 2020; Kremer et al., 2020; Mao et al., 2020; Oxley et al., 2020). Some frequently reported symptoms are not severe (such as headache, malaise, dizziness, loss of taste and smell; Mao et al., 2020), but other most severe brain conditions such as stroke and encephalitis are also common (Paterson et al., 2020; Varatharaj et al., 2020). These observations have highlighted the need to deeply describe the clinical and epidemiological characteristics of these conditions including their long-term consequences in infected individuals as well as the possible mechanisms involved.

**Figure 1:**
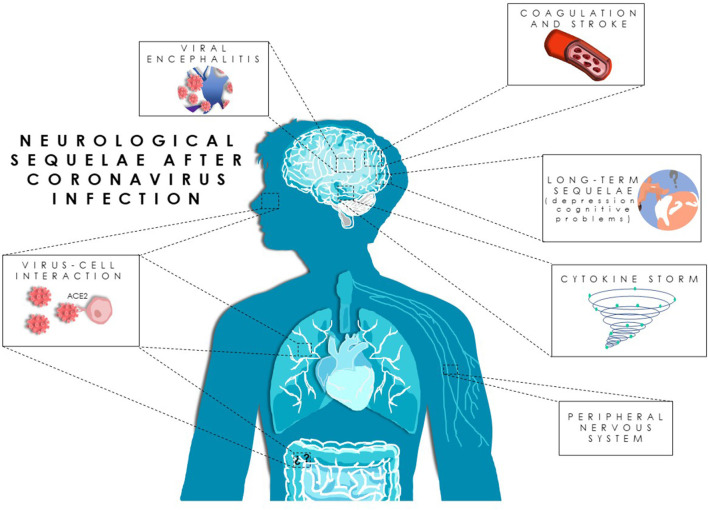
Neurological sequelae after severe acute respiratory syndrome coronavirus 2 (SARS-CoV-2) infection. Proposed pathways for SARS-CoV-2 neuroinvasion and neurological manifestations in coronavirus disease 2019 (COVID-19) patients.

This review article aims to give an overview of those neurological symptoms associated with COVID-19 (Figure 1), attempting to provide an insight beyond the acute affectation.

## Nervous System Diseases Related to SARS-CoV-2 Infection

### Viral Encephalitis (VE)

Viral encephalitis (VE) is a syndrome caused by neurotropic viruses (Tyler, 2018) characterized by altered mental status. The symptoms consist of a combination of acute fever, seizures, neurologic deficits, cerebrospinal fluid (CSF) pleocytosis, and neuroimaging and electroencephalographic (EEG) abnormalities. Clinical cases of VE in COVID-19 patients have been reported (Etemadifar et al., 2020; Moriguchi et al., 2020; Paniz-Mondolfi et al., 2020; Ye et al., 2020; Zhou F. et al., 2020), suggesting a potential invasion capacity of this virus into the Central Nervous System (CNS), as shown by other members of the Coronaviridae family (Xu et al., 2005; Morfopoulou et al., 2016; Nilsson et al., 2020). Neurological complications induced by respiratory viruses from the Coronaviridae family, such as HCoV-OC43, HCoV-229E, and SARS-CoV-1, have been already reported (Sharifian-Dorche et al., 2020).

The mechanisms by which SARS-CoV-2 causes encephalitis are poorly understood due to the low number of reported cases. It is speculated that the infection might occur after an inflammatory injury, rather than direct viral infection (Zhou Z. et al., 2020). However, given the high homology of SARS-CoV-2 with SARS-CoV-1, direct damage to the CNS cannot be ruled out. Similarly, the infection with HCoV-OC43, a coronavirus that presented a particular tropism for neurons and can produce direct neuronal death, also causes encephalitis that can be enhanced by the host immune responses (Butler et al., 2006). Intranasal administration of SARS-CoV-2 plaque-forming units in K18-hACE2 mice have demonstrated the capacity of neuroinvasion of SARS-CoV-2 causing encephalitis symptoms, including cytokine and chemokine production, leukocyte infiltration, hemorrhage, and neuronal cell death (Kumari et al., 2021). Moreover, in this mouse model, the onset of the severe disease was correlated with the maximal viral levels in the brain (Kumari et al., 2021). Also, findings in mice show that those that survived acute encephalitis induced by HCoV-OC43 infection, developed long-term sequelae, such as hypo-activity in the open field test and decreased hippocampal excitability with concomitant neural loss in CA1 and CA3 hippocampal regions (Divani et al., 2020). However, most of the reported cases of COVID-19 patients with the manifestation of encephalitis do not have detectable SARS-CoV-2 RNA in CSF samples, which does not necessarily exclude direct CNS infection (Divani et al., 2020). In fact, experience with other infections, such as tick-borne encephalitis, suggests that there is no correlation between viral load, the timing of viremia, and clinical severity (Umapathi et al., 2020). Not surprisingly, diagnostic criteria for viral encephalitis do not require demonstration of viral particles in CSF or blood (Umapathi et al., 2020). Nevertheless, diagnosis currently relies heavily on virus isolation in the CSF. Diagnosis of COVID-19-related encephalitis can be extremely challenging, as the definitive diagnosis is highly dependent on CSF virus isolation. This trouble becomes difficult in COVID-19 patients because the dissemination of SARS-CoV-2 is transient and its titer in CSF can be extremely low (Haider et al., 2020). Further studies, both, in patients and animal models, are required to precisely determine the extent of neurological sequelae of SARS-CoV-2-related encephalitis.

To summarize, the lack of continuity and consistency in encephalopathies in COVID-19 patients leaves us without a clear picture of where the neurological abnormalities may come from. Therefore, more in deep studies are necessary to clarify the exact role of SARS-CoV-2 in this disease.

### Peripheral Nervous System Disease

Coronavirus family also affects the peripheral nervous system (PNS) which could be caused directly by the virus or by the body’s innate and adaptive immune responses to infection. The Guillain-Barré syndrome (GBS) is an autoimmune neurologic disease of PNS caused by an infection leading to an autoimmune response, which produces demyelination and injury of axons. The disease symptoms begin with weakness and tingling in the extremities leading to rapidly progressive, symmetrical limb weakness, areflexia on examination, sensory symptoms, and, in some patients, facial weakness, although several variants exist (Willison et al., 2016). The first case of COVID-19 initially associated with acute GBS was reported by Zhao et al. (2020). To date, 50 GBS patients, or its variants, and COVID-19 have been reported (for review, see Katyal et al., 2020; Satarker and Nampoothiri, 2020; Sriwastava et al., 2020). The mean latency between infection and GBS symptoms ranged between 11 and 13 days (Sriwastava et al., 2020). It has been hypothesized that the various mechanisms by mean of SARS-CoV-2 trigger GBS: (i) cross-reactivity between the viral protein (viral spike (S) protein)—associated gangliosides containing sialic acid residues, including the GalNAc residue of GM1 (Ahmed et al., 2020; Baig et al., 2020; Caress et al., 2020; Dalakas, 2020; Sriwastava et al., 2020; Zhou Z. et al., 2020) and peripheral nerve gangliosides as the result of molecular mimicry. Serum ganglioside antibodies were found in 7% of described COVID-19-GBS patients (for review, see Sriwastava et al., 2020). (ii) T-cell activation and release of inflammatory mediators as cytokine storms induce nerve damage and, therefore trigger GBS in COVID-19 patients. Interestingly, none of the reported patients had positive PCR for SARS-CoV-2 in the CSF (Sriwastava et al., 2020) but the damage could be produced by the breakdown of the blood-brain barrier rather than direct intracranial viral invasion (Ahmed et al., 2020; Zhou F. et al., 2020). Although a direct effect of the virus on the PNS could not be ruled out, probably, the cytokine storm described in a proportion of the most severe COVID-19 patients triggers the neurological symptoms, including GBS, as has been proposed for some other viral infectious diseases (Chousterman et al., 2017). However, a more in-depth study would need to unequivocally demonstrate the relationship of GSB with SARS-CoV-2 infection, despite the low proportion of COVID-19 patients presenting GBS.

### Acute Cerebrovascular Disease

Acute cerebrovascular disease is caused by the blood supply disruption in the brain under ischemic or hemorrhagic conditions, such as thrombotic or embolic occlusion. The brain responds to this blood disruption altering the metabolism, the microvascular hemodynamics, and the collateral flow interactions. These responses may result in brain damage and even death (Gaddi et al., 2003; Donahue and Hendrikse, 2018). Beyond the motor impairment observed in stroke patients, the long-term neurological manifestations include depression and cognitive impairment followed by dementia, recurrent strokes, epilepsy, bleeding and also, death (Singh et al., 2018).

It is known that between 0.2 and 1% of COVID-19 patients undergo ischemic strokes (Altable and De La Serna, 2020), and it is thought to be caused by the prothrombotic effect as a consequence of the inflammatory response (for review, see Abou-Ismail et al., 2020). COVID-19 patients with a historical cerebrovascular disease (CVD), may present increased severity. Besides, patients with severe infection are more prone to display a CVD rather than the ones with less severe infection (Li et al., 2020). Moreover, other comorbidities, such as diabetes mellitus, high coagulation and hypertension, and aging enhance the CVD in COVID-19 patients (Goldberg et al., 2020; Larson et al., 2020).

There are several mechanisms by which the SARS-CoV-2 virus might cause brain stroke (Trejo-Gabriel-Galan, 2020), including: (i) invasion of vascular walls by directly joining angiotensin-converting enzyme 2 (ACE2) receptors located on the surfaces of the endothelial cells; (ii) coagulopathy associated with COVID-19: produced by the cytokine storm that increases the D-dimers levels [the fibrin degradation products found in the blood after blood clots degradation which is associated to high mortality in COVID-19 patients (Rostami and Mansouritorghabeh, 2020)]; (iii) myocardial damage with cerebral embolism: SARS-CoV-2 could damage the heart, which in turn causes a cardioembolic stroke, measured by increased troponin levels (Huang et al., 2020; Zhou F. et al., 2020); and, (iv) destabilization of a pre-existing atheroma plaque: systemic inflammation might break the fibrous cap of the atheroma and the thrombogenic material can be released to the blood, leading to a coagulation cascade and recruitment of inflammatory cells and circulating platelets (Badimon and Vilahur, 2014). However, the exact mechanism by which SARS-CoV-2 could be involved in the CVD needs further investigation.

### Cognitive Decline After Overcoming Acute SARS-CoV-2 Infection in COVID-19

Neurological effects have been associated with COVID-19 including confusion, disorientation, agitation, and drowsiness (Helms et al., 2020; Heneka et al., 2020). In fact, a total of 33% of the discharged patients presented mental alterations and motor deficiencies (Helms et al., 2020). These symptoms could be caused by the dysfunction of peripheral organs, encephalitis, systemic inflammation, and cerebrovascular alterations. These conditions would expose COVID-19 survivors at risk of long-term neurological consequences, either by aggravating a pre-existing disorder or by initiating them (Heneka et al., 2020). Therefore, it has been suggested that individuals who survive the most severe COVID-19 are at high risk to develop neurological diseases, and in particular, Alzheimer’s disease (Tejera et al., 2019). So far, it seems unlikely that the virus has a role in causing or exacerbating Parkinson’s disease, but the aggravation of specific motor and non-motor symptoms has been recently discussed (Sulzer et al., 2020). Whether or not, these complications are directly produced by the virus or indirectly enhanced by the cytokine storm displayed by the immune system or both remains unknown due to the scarcity of histopathological evidence available. Thus, it has been reported that strokes appear to be more related to hypercoagulability and endothelial injury than to the direct SARS-CoV-2 vasculitis affecting the brain (Iadecola et al., 2020). However, there is evidence of brain infection by SARS-CoV-2 (Deigendesch et al., 2020; Moriguchi et al., 2020; Paniz-Mondolfi et al., 2020) that deserves special attention in this review article.

## Potential Neuro-Invasive Mechanisms of SARS-CoV-2

Experimental and clinical studies have demonstrated a neuro-invasive potential of human and animal coronaviruses (Pennisi et al., 2020). A recent report by Mao et al. (2020) described that 36.4% of the 214 total patients with SARS-CoV-2 infection exhibited neurological symptoms, suggesting its neuro-invasive potential, especially in the most severe cases (Helms et al., 2020; Paterson et al., 2020; Varatharaj et al., 2020). Moreover, there are reports of the presence of SARS-CoV-2 in brains or CSF from COVID-19 patients (Deigendesch et al., 2020; Moriguchi et al., 2020; Paniz-Mondolfi et al., 2020).

Although the precise mechanism by which SARS-CoV-2 can reach the CNS has not yet elucidated, based on previous knowledge about the infection mechanisms of other coronaviruses, two hypotheses emerge as the potential routes of how SARS-CoV-2 enters into the brain: (i) through retrograde axonal transport; and/ or (ii) hematogenous spread from systemic to the cerebral circulation.

Within the first alternative, SARS-CoV-2 could infect the peripheral neurons in the olfactory tract and might reach the brain through retrograde axonal dissemination. The olfactory bulb is connected through the cribriform plate with the olfactory receptor neurons (van Riel et al., 2015). It is well known that ACE2 receptors are the key molecules to allow the entry of the virus into cells. These receptors are expressed not only on the epithelial cells of the mucosa (Xu et al., 2020) but also in glial cells, neurons, and in endothelial and arterial smooth muscle cells (Baig et al., 2020; Deigendesch et al., 2020; Xu and Lazartigues, 2020). This fact could enhance viral dissemination. This hypothesis was previously tested for SARS-CoV infection using a human transgenic mouse model for ACE2 receptors (Netland et al., 2008). The authors showed that the virus infected the olfactory bulb and spread reaching the brain and causing neuronal death, especially affecting those neurons located in the cardiorespiratory centers. Although this mechanism could explain the loss of smell and taste in COVID-19 patients (Giacomelli et al., 2020), the retrograde axonal transport hypothesis needs to be investigated for SARS-CoV-2.

Additionally, COVID-19 patients also present gastrointestinal alterations (Silva et al., 2020). As already described in MERS-CoV infection (Zhou et al., 2017), a new potential route for viral neuroinvasion has been proposed for SARS-CoV-2 (Esposito et al., 2020). *In vitro* experiments, using human small intestine and brain organoids, and histological characterizations for human intestine samples, have demonstrated the capacity of SARS-CoV-2 to infect the gastrointestinal tract (Lamers et al., 2020; Zhang H. et al., 2020; Silva et al., 2020; Kumari et al., 2021). This invasion may activate the enteric glial cells inducing the cytokine storm observed in COVID-19 patients (Esposito et al., 2020). Moreover, enteric glial cells are crucial regulators of gut-brain signaling and their activation has been related to the viral infection by HIV-1 Tat-associated gastrointestinal and neurological impairments (Esposito et al., 2017).

The second hypothesis is based on the hematogenous dissemination of the virus from the systemic to the cerebral circulation. In this route, the virus might extend to the brain by binding to ACE2 receptors present on the endothelial cells and smooth muscles in the cerebral microvasculature, inducing BBB disruption. This possible way is supported by the high expression of ACE2 receptors and associated proteases in the vascular endothelium, suggesting that these cells could be also targeted by the SARS-CoV-2 (Monteil et al., 2020). Besides, and based on other coronavirus studies, cytokines might be playing a fundamental role inducing neuroinflammation. In fact, one of the major manifestations in COVID-19 severe patients is the cytokine storm (Qin et al., 2020; Wang et al., 2020). It could also alter the BBB, enabling the viral entry into the brain through the hematogenous way (Pellegrini et al., 2020). Furthermore, cytokine storm in response to viral infections induces clotting in the cerebral vasculature (Mizuguchi et al., 2007) also recently described in a clinical case of a severe COVID-19 patient (Muhammad et al., 2020). For this reason, the anticoagulant medication appears as a promising treatment in severe COVID-19 patients associated with coagulopathy (Tang et al., 2020). Interestingly, SARS-CoV-2 particles have been found in brain microvascular endothelial cells in the neural niche (Paniz-Mondolfi et al., 2020). Nevertheless, the hematogenous alternative needs to be demonstrated.

Nonetheless, the exact mechanism by which SARS-CoV-2 leads to neurological symptoms is still undetermined and requires further investigations.

## Conclusion

The clinical manifestations of SARS-CoV-2 infection are in the early phase prominent in the lungs where ACE2 is highly expressed. However, apart from the lungs and intestines, ACE2 is expressed in venous and arterial endothelial cells and arterial smooth muscle cells in most of the organs (Hamming et al., 2004), which could open up for COVID-19 to become a systemic disease. The nervous system is not unfamiliar with the influence of the virus and two non-exclusive pathways of viral neuroinvasion have been postulated: (i) through retrograde axonal transport, and/or (ii) hematogenous spread from systemic to the cerebral circulation. Data on the infection of the olfactory bulb enabling access to the brain through retrograde axonal dissemination in SARS-CoV infection (Netland et al., 2008) and experiments showing the infection of choroid plexus cells in human brain organoids (Pellegrini et al., 2020) strongly suggest the existence of these two pathways. Independently of the pathways that SARS-CoV-2 uses to reach the CNS if the immune response against the virus is not contained and feedback loop mechanisms non-functional, which are otherwise exquisitely regulated under physiologic conditions, detrimental hyperinflammation/SIRS can occur. This affectation allows an aberrant deleterious response, causing a cytokine storm with severe multiorgan manifestations including those of the CNS, favoring the disruption of the BBB and the infiltration of different immune cells, such as cytotoxic T lymphocytes and monocytes with profound proinflammatory potential. This scenario is the ground to pro-atherogenic manifestations, such as CVD including stroke, but also encephalitis, affectations of the peripheral nervous system or different grades of cognitive decline after overcoming the primary SARS-CoV-2 infection. In the absence of effective antivirals, therapeutic efforts have to be paid to decrease this aberrant immune response, trying to apply immunomodulators that balance the antiviral effect of the immune system without producing and aberrant autoimmune hyper-inflammatory response, in order to decrease neurological sequelae associated to COVID-19. However, if the infection of the nervous system by SARS-CoV-2 is confirmed, a new challenge would appear in the battle against the COVID-19 disease.

## Author Contributions

RR and LR conceptualized, designed, and drafted the manuscript. IA-B, SB, GV, LC-H, EM, and ER-M were involved in the literature search and drafted the manuscript. The image was made by IA-B, following the guidelines of SB. TD and JLV critically revised and edited the manuscript. All authors contributed to the article and approved the submitted version.

## Conflict of Interest

The authors declare that the research was conducted in the absence of any commercial or financial relationships that could be construed as a potential conflict of interest.
